# A comparison of the Child Health Utility 9D and the Health Utilities Index for estimating health utilities in pediatric inflammatory bowel disease

**DOI:** 10.1007/s11136-023-03409-x

**Published:** 2023-04-01

**Authors:** Naazish S. Bashir, Thomas D. Walters, Anne M. Griffiths, Anthony Otley, Jeff Critch, Wendy J. Ungar

**Affiliations:** 1 Program of Child Health Evaluative Sciences, The Hospital for Sick Children Research Institute, Toronto, ON Canada; 2 Division of Gastroenterology, Hepatology and Nutrition, The Hospital for Sick Children, Toronto, ON Canada; 3 Department of Paediatrics, University of Toronto, Toronto, ON Canada; 4 Departments of Paediatrics and Medicine, Dalhousie University, Halifax, NS Canada; 5 Division of Gastroenterology & Nutrition, IWK Health Centre, Halifax, NS Canada; 6 Department of Pediatrics, Faculty of Medicine, Janeway Children’s Health and Rehabilitation Centre, Memorial University, St. John’s, NF Canada; 7 Institute of Health Policy, Management and Evaluation, University of Toronto, Toronto, ON Canada

**Keywords:** Health utilities, CHU9D, HUI, Crohn’s disease, Ulcerative colitis, Child health

## Abstract

**Purpose:**

Health utilities are challenging to ascertain in children and have not been studied in pediatric Crohn’s disease (CD) and ulcerative colitis (UC). The objective was to assess discriminative validity by comparing utilities elicited using the Child Health Utility-9 Dimension (CHU9D) to the Health Utilities Index (HUI) across multiple disease activity scales in pediatric UC and CD.

**Methods:**

Preference-based instruments were administered to 188 children with CD and 83 children with UC aged 6 to 18 years. Utilities were calculated using CHU9D adult and youth tariffs, and HUI2 and HUI3 algorithms in children with inactive (quiescent) and active (mild, moderate, and severe) disease. Differences between instruments, tariff sets and disease activity categories and were tested statistically.

**Results:**

In CD and UC, all instruments detected significantly higher utilities for inactive compared to active disease (*p* < 0.05). Mean utilities for quiescent disease ranged from 0.810 (SD 0.169) to 0.916 (SD 0.121) in CD and from 0.766 (SD 0.208) to 0.871 (SD 0.186) in UC across instruments. Active disease mean utilities ranged from 0.694 (SD 0.212) to 0.837 (SD 0.168) in CD and from 0.654 (SD 0.226) to 0.800 (SD 0.128) in UC.

**Conclusion:**

CHU9D and HUI discriminated between levels of disease activity in CD and UC regardless of the clinical scale used, with the CHU9D youth tariff most often displaying the lowest utilities for worse health states. Distinct utilities for different IBD disease activity states can be used in health state transition models evaluating the cost-effectiveness of treatments for pediatric CD and UC.

**Supplementary Information:**

The online version contains supplementary material available at 10.1007/s11136-023-03409-x.

## Plain English Summary

Inflammatory Bowel Disease (IBD) is an ongoing childhood condition that can be physically painful and greatly reduces the quality-of-life of affected children. It can be difficult to measure quality-of-life in children, particularly in very young children. Being able to measure quality-of-life for different levels of disease is important to understand the effectiveness of new treatments. Using a generic common measure of quality-of-life enables a comparison of health improvement across different patient populations. This is very useful for decision-makers considering value-for-money when allocating healthcare budgets. The aim was to compare different questionnaires to see which was best able to reflect changes in quality-of-life when IBD worsens in a group of 271 children with IBD. While all questionnaires were able to pick up quality-of-life differences when disease activity changed, the Child Health Utility-9 Dimensions (CHU9D) performed the best. The findings can be used by researchers searching for the right questionnaire to use in studies that look at value-for-money of new treatments for children with IBD.

## Introduction

Ulcerative colitis (UC) and Crohn’s disease (CD), collectively referred to as inflammatory bowel disease (IBD), are a class of chronic gastrointestinal diseases characterized by periods of unpredictable flares of inflammation of the gastrointestinal tract, abdominal pain, diarrhea, fatigue and weight loss, and periods of symptomatic remission [1]. Pediatric IBD is of particular concern because the incidence is increasing [2–4], growth may be affected [1, 5] and it can have significant quality-of-life impacts [6–9]. With an increase in costly IBD treatments such as biologics, there is a need for economic evaluations to inform funding. However, measuring preference-based health-related quality-of-life (HRQOL) to generate health state utilities to calculate quality-adjusted life years (QALYs) for use in economic evaluation is challenging in children. In addition to challenges children experience in comprehending abstract concepts, HRQOL attributes featured in adult instruments may not be applicable to children. The validity of applying existing preference-based HRQOL instruments in children has been questioned [10–13]. Head-to head comparisons in pediatric patient populations are lacking [14, 15]. Further, whether the set of utility weights that underly HRQOL classification systems should be derived from adults valuing pediatric states or directly from children continues to be debated [10, 16].

The Health Utilities Index (HUI) has been used in children and adults [17] and generates utilities using the HUI Mark 2 (HUI2) or Mark 3 (HUI3). The HUI2 has 7 dimensions: Sensation, Mobility, Emotion, Cognition, Self-Care, Pain and Fertility, and the HUI3 has 8 dimensions: Vision, Hearing, Speech, Ambulation, Dexterity, Emotion, Cognition and Pain [17, 18]. The underlying utility weights for HUI2 and HUI3 were developed with adults. While the HUI has been used in numerous patient populations and across several age groups [18], it had not been used in pediatric IBD [7]. The Child Health Utility 9D (CHU9D) was developed in 2009 specifically for children and with children [19, 20]. It features a classification system of nine dimensions relevant to child health: Worried, Sad, Pain, Tired, Annoyed, Schoolwork, Sleep, Daily routine, and Activities [21]. Sets of utility weights obtained from Australian adults or adolescents are available [22, 23].

An earlier study by our team found the CHU9D to be valid and reliable in pediatric CD and UC with moderate correlations observed between CHU9D, HUI2, and HUI3 utilities [24]. A Moderate to strong correlations between the CHU9D, HUI2, HUI3 and the disease-specific IMPACT-III or the generic PedsQL HRQOL measures were observed [25]. Multiple clinical measures may be used to determine disease activity in pediatric IBD. It’s critical to examine and compare utilities associated with different measures to inform health state modeling used in cost-effectiveness analysis. The objective was to directly compare CHU9D using adult and youth tariffs to the HUI2 and HUI3 for determining health state utilities for distinct disease activity levels defined by different clinical scales in children with UC and CD. Such information is vital to assess the discriminant validity of preference-based HRQOL instruments and inform the choice of measures for use in children. This work also aims to derive utilities for health states for cost-effectiveness analysis in pediatric IBD.

## Methods

The study was approved by the Research Ethics Boards of the Hospital for Sick Children in Toronto, ON (#1000039604), the IWK Health Centre in Halifax, NS (#1015558), and the Janeway Children’s Health and Rehabilitation Centre in St. John’s NL (#13.229). The study was performed in accordance with the ethical standards as laid down in the 1964 Declaration of Helsinki and its later amendments or comparable ethical standards. All parent participants provided informed consent and children provided informed assent.

### Study design and participants

Participants were recruited from the Canadian Children Inflammatory Bowel Disease Network (CIDsCaNN) (https://cidscann.ca), a repeated measures observational cohort study of Canadian children with newly diagnosed IBD [26]. Children were treated at the discretion of the attending clinicians. Children aged 6 to 18 years without co-morbidities were recruited between February 2014 and December 2018.

### Measures

As indirect approaches to utility estimation, the HUI and CHU9D consist of a classification system and an underlying tariff set. The instrument attributes were derived from qualitative research to capture the construct of HRQOL [17, 27]. Underlying tariff sets used to score the instrument are constructed by eliciting utilities for a wide range of health states from the public using standard gamble, time-trade-off or a discrete choice approach [17, 23, 27, 28]. A utility function is then derived to enable calculation of a summary utility score with interval scale properties for any given health state from 0 (death) to 1.0 (perfect health). Details are provided in Online Resource 1. The weighted Pediatric Crohn’s Disease Activity Index (wPCDAI) [29, 30] and the Pediatric Ulcerative Colitis Activity Index (PUCAI) [31–33] are widely accepted disease activity measures for children with CD and UC, respectively. The disease activity of each participant was categorized as none (quiescent), mild, moderate, or severe based on PUCAI and wPCDAI numerical score cut-offs [29, 30, 32, 33]. For this paper, disease activity labeled as ‘none,’ ‘remission’ or ‘quiescent’ is referred to as quiescent. A Physician Global Assessment (PGA) of disease activity also categorized disease activity as quiescent, mild, moderate, severe, or fulminant. As a global measure, the PGA is based on the physician’s determination of a patient’s health, requires less data and relies less on objective measures than the wPCDAI and the PUCAI. Thus the PGA rating may be more readily attainable [30, 31]. The wPCDAI and PUCAI have been correlated with the (PGA [30, 31, 34]. Disease activity was grouped into two categories: inactive (quiescent) and active (mild, moderate, severe or fulminant). This dichotomization recognizes that an important treatment difference lies between active disease, such as relapse with varying levels of disease activity, versus inactive disease, such as remission, where medications may be withdrawn. This facilitates the option of creating two distinct disease activity states with corresponding health utilities for use in health state transition modeling.

### Data collection

The CHU9D and HUI were administered electronically in English via REDCap [35] at repeat assessments and were self-completed or self-assessed but interviewer-assisted, depending on the preference of the participant or caregiver. IBD-related disease activity was assessed at the time of CHU9D and HUI completion by study physicians using the PGA, and the wPCDAI [30] or PUCAI [33] for CD and UC patients, respectively.

### Analysis

This study aimed to assess and compare the discriminant validity [36, 37] of the CHU9D and HUI by examining the ability of each tool and tariff set to distinguish between levels of disease activity as defined by the PGA, the wPCDAI and PUCAI. For each participant, the first available pair of complete date-matched CHU9D and HUI questionnaires administered following disease diagnosis was analyzed. As no US or Canadian tariffs were available at the time of analysis, CHU9D utility weights were calculated using Australian adult tariffs and Australian adolescent tariffs with scoring algorithms provided in STATA by the developers [23, 38]. HUI utility weights were calculated based on HUI2 and HUI3 algorithms provided by Health Utilities Inc. (http://healthutilities.com/) under licence. The CHU9D and HUI algorithms were coded and all instruments scored using the R statistical software program (v. 4.0.0) [39]. The optional HUI2 “Fertility” attribute was omitted for this pediatric population.

Statistical analysis was conducted using R (v. 4.0.0) [39]. CD and UC patient data were analysed separately. Descriptive statistics were compiled using the Table One package in R [40]. Chi-square test, *t *test (for continuous variables), and Kruskal–Wallis rank-sum tests were used for demographic comparisons between sexes. The Shapiro–Wilk normality test was performed to examine the distribution of the CHU9D utilities calculated with adult and youth tariffs and for the HUI2 and HUI3 health utilities. The null hypothesis was rejected, and normality could not be assumed. Therefore, Kruskal–Wallis rank-sum tests were used to compare utilities between instruments. Medians and interquartile ranges (IQR) were calculated for all estimates in additions to means and standard deviations (SD). CHU9D (adult and youth tariffs), HUI2 and HUI3 mean and median utilities were determined by disease activity level based on wPCDAI, PUCAI and PGA and for the dichotomized categories of active and inactive disease. Spearman correlations were determined between PGA and wPCDAI scores in CD subjects and between PGA and PUCAI scores in UC subjects. A Wilcoxon Rank Sum Test continuity correction with Bonferroni adjustment using the R ‘stats’ package [39] was conducted to compare CHU9D health utilities using adult and youth tariffs, HUI2 and HUI3 utilities between males and females, and to compare utilities between different disease activity levels as assessed by the PGA, wPCDAI and PUCAI. Box plots of mean health utilities for different activity levels were plotted using the R ‘ggpubr’ package [41].

## Results

### Sample characteristics

A total of 312 children with CD who consented to CIDsCaNN were eligible to participate in this sub-study. Of these, 116 (37.2%) were excluded due to the unavailability of date-matched CHU9D, HUI and clinical measures and 8 (< 1%) were excluded due to the use of proxy respondents, resulting in a CD sample of 271. A total of 138 CIDsCaNN participants with UC were eligible. Of these, 53 (38.4%) were excluded due to the unavailability of date-matched questionnaires and 2 (< 1%) were excluded due to the use of proxy respondents, resulting in a UC sample of 83. There were no statistically significant differences in demographic and health characteristics between males and females with CD and with UC (Table 1). At time of first CHU9D-HUI paired assessment, 39.9% of CD participants had quiescent (inactive) disease and 49.5% had mild, moderate, or severe (active) disease activity based on wPCDAI scores, and 55.3% had active disease based on PGA. In UC, 55.4% had inactive and 43.4% had active disease based on PUCAI scores, and 47.0% had active disease based on PGA. The Spearman correlation between the PGA and wPCDAI scores was 0.85 (*p* < 0.05) in CD and was 0.91 (*p* < 0.05) between the PGA and PUCAI scores in UC, indicating strong correlation between the clinical health assessment scales.

**Table 1:** Study sample characteristics for children with Crohn’s disease and ulcerative colitis

Characteristic	CD			UC		
	Males	Females	Total CD	Males	Females	Total UC
n (%)	120 (63.8)	68 (36.2)	188	34 (41.0)	49 (59.0)	83
Mean age, years (SD)^a^	13.8 (2.3)	14.1 (2.4)	13.9 (2.4)	14.3 (2.3)	14.0 (2.9)	14.1 (2.6)
Age range (years)^a^	7.3–17.8	7.6–18.1	7.3–18.1	9.4–18.0	6.5–18.1	6.5–18.1
Ethnicity (%)						
Caucasian	71 (59.2)	45 (66.2)	116 (61.7)	21 (61.8)	33 (67.3)	54 (65.1)
Mixed	13 (10.8)	7 (10.3)	20 (10.6)	2 (5.9)	4 (8.2)	3 (3.6)
South Asian	13 (10.8)	2 (2.9)	15 (8.0)	7 (20.6)	7 (14.3)	14 (16.9)
East and Southeast Asian	3 (2.5)	1 (1.5)	4 (2.1)	0 (0.0)	0 (0.0)	0 (0.0)
Caribbean	2 (1.7)	3 (4.4)	5 (2.7)	0 (0.0)	1 (2.0)	1 (1.2)
West Central Asian and Middle Eastern	2 (1.7)	2 (2.9)	4 (2.1)	1 (2.9)	3 (6.1)	4 (4.8)
Latin, Central and South American	1 (0.8)	2 (2.9)	3 (1.6)	0 (0.0)	0 (0.0)	0 (0.0)
African	5 (4.2)	2 (2.9)	7 (1.6)	2 (5.9)	1 (2.0)	3 (0.0)
Other or Unknown	10 (8.3)	4 (3.3)	14 (7.4)	1 (2.9)	0 (0.0)	1 (1.2)
Clinical Site (%)						
Toronto	91 (75.8)	54 (73.5)	141 (75.0)	39 (85.3)	42 (85.7)	71 (86.0)
Halifax	29 (24.2)	15 (22.1)	44 (23.4)	5 (14.7)	6 (12.2)	11 (13.3)
St. John’s	0 (0.0)	3 (4.4)	3 (1.6)	0 (0.0)	1 (2.0)	1 (1.2)
PGA (%)						
Quiescent	56 (46.7)	28 (41.2)	84 (44.7)	17 (50.0)	27 (55.1)	44 (53.0)
Mild	39 (32.5)	15 (22.1)	54 (28.7)	12 (35.3)	9 (18.4)	21 (25.3)
Moderate	22 (18.3)	20 (29.4)	42 (22.3)	2 (5.9)	7 (14.3)	9 (10.8)
Severe	3 (2.5)	5 (7.4)	8 (4.3)	3 (8.8)	5 (10.2)	8 (9.6)
Fulminant	0 (0.0)	0 (0.0)	0 (0.0)	0 (0.0)	1 (2.0)	1 (1.2)
wPCDAI or PUCAI (%)						
Quiescent	51 (42.5)	24 (35.3)	75 (39.9)	18 (52.9)	28 (57.1)	46 (55.4)
Mild	30 (25.0)	22 (32.4)	52 (27.7)	8 (23.5)	9 (18.5)	17 (20.5)
Moderate	13 (10.8)	4 (5.9)	17 (9.0)	3 (8.8)	7 (14.3)	10 (12.0)
Severe	11 (9.2)	13 (19.1)	24 (12.8)	4 (11.8)	5 (10.2)	9 (10.8)
Missing (Unknown)	15 (12.5)	5 (7.4)	20 (10.6)	1 (2.9)	0 (0.0)	1 (1.2)

^a^Age at time of assessment is reported

*CD* Crohn’s disease; *PGA* Physician Global Assessment; *PUCAI* Pediatric Ulcerative Colitis Activity Index; *SD* standard deviation; *UC* ulcerative colitis; *wPCDAI* weighted Pediatric Crohn’s Disease Activity Index

### Overall utilities

**Fig. 1:**
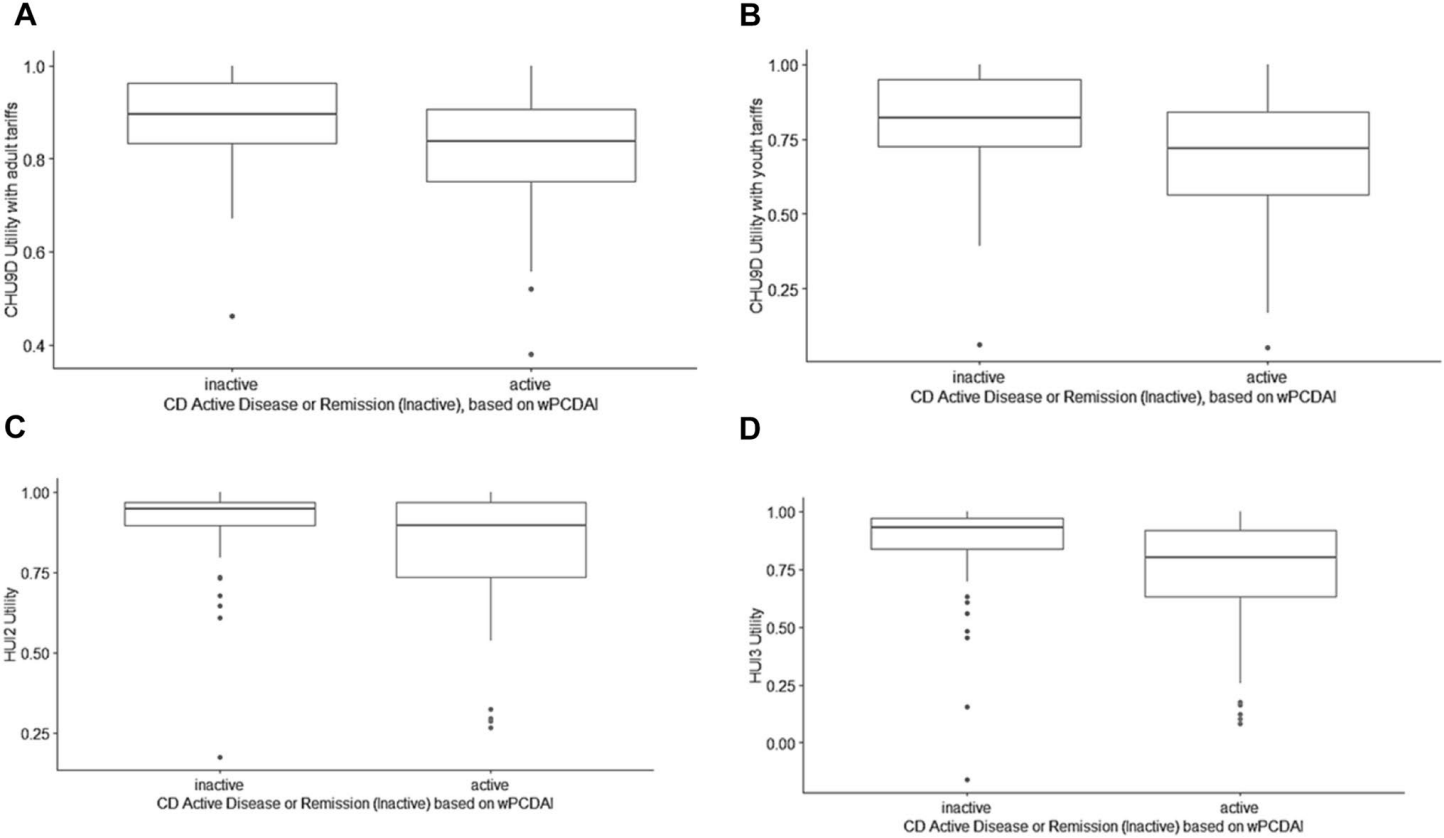
Boxplots comparing utilities in Crohn’s disease patients in inactive and active disease categories as determined by the weighted Pediatric Crohn’s Disease Activity Index (wPCDAI) for: **A** CHU9D adult tariffs, **B** CHU9D youth tariffs, **C** HUI2, and **D** HUI3. There was a significant difference between utilities in active and inactive disease (*p *< 0.05)

**Fig. 2:**
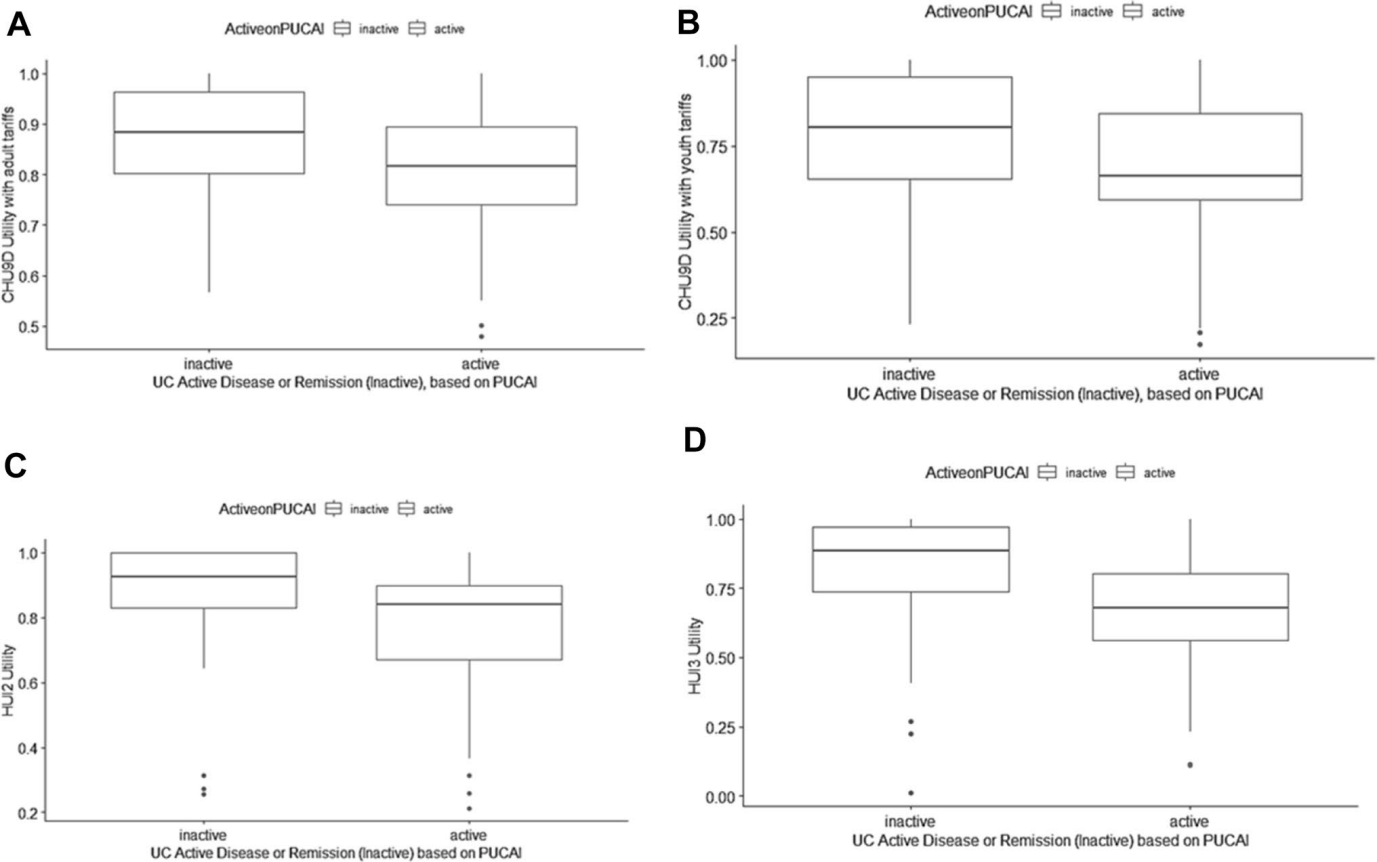
Boxplots comparing utilities in ulcerative colitis patients in inactive and active disease categories as determined by the Pediatric Ulcerative Colitis Activity Index (PUCAI) for: **A** CHU9D adult tariffs, **B** CHU9D youth tariffs, **C** HUI2, and **D** HUI3. There was a significant difference between utilities in active and inactive disease (*p* < 0.05)

As seen in Table 2, for the CD sample as a whole, mean/median utilities in CD ranged from 0.757/0.792 to 0.873/0.926 across instruments. All instruments demonstrated lower utilities with increasingly active disease states. CHU9D youth tariff utilities consistently exhibited the lowest mean and median scores while the HUI2 almost always exhibited the highest overall mean and median scores. For UC as a whole, mean/median utilities ranged from 0.719/0.737 to 0.833/0.888. All instruments generally demonstrated lower utilities with increasingly active disease except where small sample sizes resulted in unstable estimates. CHU9D utilities calculated with youth tariffs were consistently lower than those calculated with adult tariffs. CHU9D youth tariff utilities most often exhibited the lowest and the adult tariff the highest mean and median scores across instruments and activity levels. Mean/median utilities were significantly lower in UC compared to CD for each instrument (*p* < 0.05). The observed differences between CHU9D adult and youth tariffs, HUI2 and HUI3 utilities for inactive versus active disease were statistically significant in CD (Fig. 1) and UC (Fig. 2).

**Table 2:** Comparison of CHU9D, HUI2 and HUI3 utilities in CD and UC patients by disease activity level

	Disease Activity Level	n	CHU9D (adult tariffs)		CHU9D (youth tariffs)		HUI2		HUI3	
			Mean (SD)	Median (IQR)	Mean (SD)	Median (IQR)	Mean (SD)	Median (IQR)	Mean (SD)	Median (IQR)
CD (all categories)		188	0.855 (0.116)	0.877 (0.156)	0.757 (0.200)	0.792 (0.238)	0.873 (0.154)	0.926 (0.127)	0.811 (0.214)	0.879 (0.214)
CD PGA	Quiescent	84	0.889 (0.101)	0.903 (0.142)	0.822 (0.170)	0.854 (0.251)	0.916 (0.121)	0.947 (0.103)	0.882 (0.184)	0.931 (0.134)
	Mild	54	0.862 (0.102)	0.879 (0.127)	0.767 (0.174)	0.801 (0.217)	0.888 (0.104)	0.896 (0.125)	0.813 (0.163)	0.859 (0.214)
	Moderate	42	0.789 (0.132)	0.834 (0.173)	0.638 (0.224)	0.719 (0.331)	0.782 (0.219)	0.872 (0.257)	0.693 (0.267)	0.801 (0.283)
	Severe	8	0.794 (0.107)	0.819 (0.061)	0.631 (0.206)	0.676 (0.115)	0.785 (0.108)	0.809 (0.169)	0.662 (0.191)	0.685 (0.127)
	Fulminant	0	NA	NA	NA	NA	NA	NA	NA	NA
CD wPCDAI	Quiescent	75	0.883 (0.100)	0.896 (0.129)	0.810 (0.169)	0.821 (0.223)	0.907 (0.123)	0.947 (0.072)	0.866 (0.190)	0.931 (0.134)
	Mild	52	0.863 (0.098)	0.881 (0.119)	0.769 (0.167)	0.793 (0.200)	0.870 (0.160)	0.917 (0.132)	0.799 (0.203)	0.859 (0.243)
	Moderate	17	0.803 (0.098)	0.819 (0.114)	0.652 (0.185)	0.693 (0.208)	0.809 (0.175)	0.848 (0.173)	0.715 (0.245)	0.801 (0.247)
	Severe	24	0.741 (0.147)	0.772 (0.194)	0.562 (0.250)	0.598 (0.341)	0.756 (0.191)	0.757 (0.227)	0.648 (0.247)	0.696 (0.253)
UC (all categories)		83	0.833 (0.126)	0.839 (0.150)	0.719 (0.218)	0.737 (0.288)	0.813 (0.211)	0.888 (0.200)	0.742 (0.237)	0.781 (0.292)
UC PGA	Quiescent	44	0.864 (0.113)	0.883 (0.158)	0.778 (0.195)	0.803 (0.304)	0.871 (0.186)	0.936 (0.153)	0.821 (0.226)	0.890 (0.237)
	Mild	21	0.802 (0.117)	0.820 (0.068)	0.652 (0.210)	0.681 (0.179)	0.763 (0.218)	0.841 (0.144)	0.696 (0.174)	0.685 (0.207)
	Moderate	9	0.778 (0.087)	0.790 (0.105)	0.610 (0.157)	0.612 (0.163)	0.644 (0.233)	0.674 (0.285)	0.555 (0.221)	0.582 (0.217)
	Severe	8	0.801 (0.211)	0.864 (0.289)	0.682 (0.338)	0.766 (0.470)	0.800 (0.211)	0.872 (0.243)	0.646 (0.315)	0.797 (0.388)
	Fulminant	1	0.894 (NA)	0.894 (NA)	0.852 (NA)	0.852 (NA)	0.937 (NA)	0.937 (NA)	0.719 (NA)	0.719 (NA)
UC PUCAI	Quiescent	46	0.858 (0.119)	0.883 (0.160)	0.766 (0.208)	0.803 (0.296)	0.866 (0.183)	0.926 (0.172)	0.811 (0.229)	0.885 (0.235)
	Mild	18	0.804 (0.116)	0.817 (0.137)	0.658 (0.210)	0.673 (0.323)	0.780 (0.220)	0.845 (0.160)	0.713 (0.189)	0.697 (0.236)
	Moderate	10	0.793 (0.138)	0.818 (0.0361)	0.643 (0.229)	0.657 (0.139)	0.751 (0.247)	0.870 (0.259)	0.659 (0.227)	0.688 (0.185)
	Severe	9	0.802 (0.155)	0.805 (0.172)	0.675 (0.251)	0.622 (0.276)	0.682 (0.237)	0.674 (0.309)	0.552 (0.268)	0.582 (0.431)

*CD* Crohn’s disease; *IQR* interquartile range; *PGA* Physician Global Assessment; *PUCAI* Pediatric Ulcerative Colitis Activity Index; *SD* standard deviation; *UC* ulcerative colitis; *wPCDAI* weighted Pediatric Crohn’s Disease Activity Index

### CD

In CD, mean/median utilities ranged from 0.810/0.821 to 0.916/0.947 for quiescent disease and from 0.562/0.598 to 0.794/0.819 for severe activity across instruments and scales (Table 2). When disease activity was defined by the PGA, quiescent disease utilities were significantly greater than mild activity for HUI3 alone (*p* < 0.05). Quiescent disease utilities were significantly greater than moderate activity utilities with the CHU9D adult and youth tariffs (*p* < 0.05) but not with HUI2 or HUI3. Quiescent disease utilities were significantly greater than severe activity utilities for CHU9D (youth tariff) and for HUI2 and HUI3 (*p* < 0.05). When disease activity was based on the wPCDAI, quiescent or mild activity utilities were significantly greater than severe utilities for all tariff sets and instruments (*p* < 0.05). There were no significant differences between mild and moderate disease activity utilities.

The means and medians of utilities from CHU9D adult and youth tariffs, HUI2 and HUI3 for inactive and active disease states in CD and UC are presented in Table 3. Mean/median utilities for active CD ranged between 0.694/0.720 and 0.837/0.896 across all utility instruments. Significantly greater utilities were observed for inactive compared to active states for all severity scales and utility instruments (*p* < 0.05). No statistically significant differences were observed in utilities between PGA and wPCDAI definitions of active disease; similar utilities for PGA and wPCDAI indicates overlap across clinical definitions and substantiates the correlation between scales.

**Table 3:** Comparison of CHU9D, HUI2 and HUI3 utilities in CD and UC patients by inactive and active disease

	Disease Activity Category	n	CHU9D (adult tariffs)		CHU9D (youth tariffs)		HUI2		HUI3	
			Mean (SD)	Median (IQR)	Mean (SD)	Median (IQR)	Mean (SD)	Median (IQR)	Mean (SD)	Median (IQR)
CD category based on PGA	Inactive Disease	84	0.889 (0.101)	0.903 (0.142)	0.822 (0.170)	0.854 (0.251)	0.916 (0.121)	0.947 (0.103)	0.882 (0.184)	0.931 (0.134)
	Active Disease	104	0.827 (0.120)	0.853 (0.133)	0.704 (0.207)	0.739 (0.251)	0.837 (0.168)	0.896 (0.202)	0.753 (0.221)	0.828 (0.220)
CD category based on wPCDAI	Inactive Disease	75	0.883 (0.100)	0.896 (0.129)	0.810 (0.169)	0.821 (0.223)	0.907 (0.123)	0.947 (0.072)	0.866 (0.190)	0.931 (0.134)
	Active Disease	93	0.821 (0.123)	0.838 (0.154)	0.694 (0.212)	0.720 (0.28)	0.829 (0.176)	0.896 (0.232)	0.745 (0.229)	0.801 (0.286)
UC category based on PGA	Inactive Disease	44	0.864 (0.113)	0.883 (0.158)	0.778 (0.195)	0.803 (0.304)	0.871 (0.186)	0.936 (0.153)	0.821 (0.226)	0.890 (0.237)
	Active Disease	39	0.798 (0.132)	0.817 (0.156)	0.654 (0.226)	0.664 (0.281)	0.747 (0.221)	0.824 (0.227)	0.654 (0.219)	0.681 (0.244)
UC category based on PUCAI	Inactive Disease	46	0.858 (0.119)	0.883 (0.16)	0.766 (0.208)	0.803 (0.296)	0.866 (0.183)	0.926 (0.172)	0.811 (0.229)	0.885 (0.235)
	Active Disease	37	0.800 (0.128)	0.816 (0.153)	0.658 (0.219)	0.664 (0.25)	0.748 (0.229)	0.841 (0.227)	0.659 (0.224)	0.681 (0.240)

*CD* Crohn’s disease; *IQR* interquartile range; *PGA* Physician Global Assessment; *PUCAI* Pediatric Ulcerative Colitis Activity Index; *SD* standard deviation; *UC* ulcerative colitis; *wPCDAI* weighted Pediatric Crohn’s Disease Activity Index

### UC

In UC, mean/median quiescent utilities ranged from 0.766/0.803 to 0.871/0.936 and from 0.552/0.582 to 0.802/0.805 for severe activity across all instruments and scales (Table 2). The smaller UC sample size was associated with wider ranges of utilities within activity levels across instruments. No significant differences in utilities were observed between quiescent, mild, or moderate activity compared to severe activity for CHU9D adult and youth tariffs with the PGA and PUCAI. For the PGA, quiescent disease utilities were significantly greater than mild activity for HUI3 (*p* < 0.05) and were significantly greater than moderate activity for HUI2 and HUI3 (*p* < 0.05). There were no significant differences in utilities between PUCAI activity levels for HUI2, but quiescent disease utilities were significantly greater than severe activity utilities for the HUI3 (*p* < 0.05). Very small sample sizes (≤ 10) in the moderate and severe groups resulted in unstable estimates.

Utilities for inactive UC were significantly greater than active disease utilities across all instruments and scales (*p* < 0.05) (Table 3). Mean/median utilities for active disease ranged from 0.654/0.664 to 0.800/0.816. As in CD, comparable utilities for PGA and PUCAI disease activity levels for each health utility instrument reflect the correlation between scales.

No statistically significant differences between utilities for males and females derived from CHU9D adult tariffs, CHU9D youth tariffs, HUI2 or HUI3 were observed in CD (Table 4). In UC, CHU9D utilities calculated with adult and with youth tariffs were significantly higher for males compared to females (*p* < 0.02). Differences in utilities between males and females with UC were not statistically significant for HUI2 or HUI3.

**Table 4:** Comparison of CHU9D, HUI2 and HUI3 utilities in CD and UC patients stratified by sex and disease activity level

	Disease Activity Level	n (M)	n (F)	CHU9D (adult tariffs)		CHU9D (youth tariffs)		HUI2		HUI3	
				Males	Females	Males	Females	Males	Females	Males	Females
				Mean (SD)	Mean (SD)	Mean (SD)	Mean (SD)	Mean (SD)	Mean (SD)	Mean (SD)	Mean (SD)
CD wPCDAI	Quiescent	51	24	0.891 (0.106)	0.867 (0.087)	0.826 (0.175)	0.777 (0.153)	0.906 (0.137)	0.909 (0.086)	0.855 (0.224)	0.892 (0.079)
	Mild	30	22	0.870 (0.102)	0.854 (0.094)	0.785 (0.170)	0.746 (0.163)	0.877 (0.150)	0.859 (0.175)	0.809 (0.188)	0.785 (0.225)
	Moderate	13	4	0.816 (0.096)	0.762 (0.105)	0.686 (0.172)	0.541 (0.206)	0.827 (0.192)	0.751 (0.093)	0.731 (0.267)	0.665 (0.173)
	Severe	11	13	0.728 (0.170)	0.751 (0.131)	0.547 (0.270)	0.575 (0.242)	0.755 (0.206)	0.757 (0.186)	0.657 (0.251)	0.640 (0.253)
UC PUCAI	Quiescent	18	28	0.877 (0.134)	0.846 (0.109)	0.794 (0.294)	0.748 (0.185)	0.883 (0.179)	0.855 (0.188)	0.844 (0.214)	0.789 (0.240)
	Mild	9	9	0.849 (0.091)	0.759 (0.126)	0.736 (0.181)	0.581 (0.218)	0.832 (0.246)	0.728 (0.192)	0.793 (0.194)	0.633 (0.155)
	Moderate	3	7	0.884 (0.101)	0.754 (0.139)	0.806 (0.178)	0.573 (0.222)	0.923 (0.074)	0.677 (0.261)	0.818 (0.100)	0.591 (0.237)
	Severe	4	5	0.871 (0.128)	0.746 (0.164)	0.778 (0.230)	0.593 (0.259)	0.700 (0.248)	0.668 (0.257)	0.568 (0.238)	0.540 (0.317)
CD category based on wPCDAI	Inactive Disease	51	24	0.891 (0.127)	0.867 (0.087)	0.826 (0.175)	0.777 (0.153)	0.906 (0.137)	0.909 (0.086)	0.855 (0.224)	0.892 (0.079)
	Active Disease	54	39	0.828 (0.106)	0.810 (0.117)	0.713 (0.212)	0.668 (0.212)	0.840 (0.176)	0.814 (0.177)	0.759 (0.226)	0.725 (0.235)
UC category based on PUCAI	Inactive Disease	18	28	0.877 (0.134)	0.846 (0.109)	0.794 (0.242)	0.748 (0.185)	0.883 (0.179)	0.855 (0.188)	0.844 (0.214)	0.789 (0.240)
	Active Disease	16	21	0.861 (0.096)	0.755 (0.132)	0.759 (0.182)	0.581 (0.217)	0.816 (0.226)	0.697 (0.222)	0.742 (0.209)	0.597 (0.219)

*CD* Crohn’s disease; *IQR* interquartile range; *PGA* Physician Global Assessment; *PUCAI* Pediatric Ulcerative Colitis Activity Index; *SD* standard deviation; *UC* ulcerative colitis; *wPCDAI* weighted Pediatric Crohn’s Disease Activity Index

## Discussion

Despite being generic, all HRQOL instruments and tariff sets discriminated moderately well between quiescent, mild, moderate and severe disease activity in CD and UC. Compared to the adult tariff set, CHU9D youth tariff utilities were consistently lower and displayed a greater range across disease activity levels for CD and UC. Similarly, the HUI3 displayed lower utilities and a wider range across disease activity levels in CD and UC compared to the HUI2.

While specific utilities for pediatric CD and UC are lacking, a meta-analysis reported mean utilities of 0.860 with the HUI2 and 0.882 with the HUI3 for digestive system disorders other than IBD, such as liver diseases, gastric ulcer, and other disorders [42]. Combined pediatric chronic diseases had mean utilities of 0.924 using a standard gamble (SG), 0.884 using the HUI2 and 0.834 with the HUI3 [42]. A meta-analysis of eleven adult CD studies determined a mean utility of 0.8403, 95% CI (0.8012, 0.8794) for remission, 0.7533, 95% CI (0.6887, 0.8178) for active disease, 0.8619, 95% CI (0.8016, 0.9223) for mild disease, 0.7318, 95% CI (0.6271 0.8364) for moderate disease and 0.5102, 95% CI (0.3554, 0.6650) for severe disease [43], values comparable to utilities observed in the present analysis. The results suggest that similar to adults, children with IBD can experience a range of utilities across clinically meaningful disease activity levels. A meta-analysis of 15 adult UC studies demonstrated a mean utility of 0.8726, 95% CI (0.8457, 0.8995) for remission, 0.6992, 95% CI (0.5847, 0.8136) for active disease, 0.7834 95% CI (0.7265 0.8403) for mild disease, 0.6969 95% CI (0.3959 0.9978) for moderate disease, and 0.7059 95% CI (0.5065 0.9054) for severe disease [43]. The present study demonstrated comparable results to adults for remission, but lower utilities for more active disease, however active disease utilities fell within the 95% confidence interval reported for adults.

Assigning utilities to health states can be complicated by the existence of multiple clinical measures used to describe disease activity. The wPCDAI has been found to perform better in measuring CD disease activity than other versions of the PCDAI and the Harvey-Bradshaw Index (HBI), developed for use in adult patients [30, 44]. In the present study, correlations between the PGA and clinical measures of disease activity for CD and UC exceeded 0.85, and the ability of the preference-based instruments to distinguish between PGA-defined disease activity levels suggest that when laboratory and other objective clinical data are unavailable, PGA utilities can be used in economic modeling. Dichotomizing disease activity as active or inactive may further facilitate modeling when a variety of scales are used or when data needed for finer disease activity stratification are missing. It’s important to note that multi-item measures of current disease activity may not incorporate how a patient’s prior disease experience or duration of disease influences their health state preferences. For example, long-term healing and achieving stable disease may reduce anxiety, fatigue, and pain which may be observed as improvement in HRQOL over time [45, 46].

A common challenge is that while all instruments aim to capture the construct of HRQOL, they may return different utilities for the same health state. These discrepancies may be due to the different domains, classification systems and underlying weights of each instrument. This raises the issue of comparability and interchangeability of pediatric health utility instruments with each other and with adult instruments. It must also be acknowledged that HRQOL in children differs from adults, and also differs within pediatric age groups from neonate to adolescent [47–50]. While the HUI2 and HUI3 have long been used in pediatric as well as adult populations, the CHU9D was designed exclusively as a tool for children aged 7 to 17 years [28, 51, 52]. Our previous research reported correlations of 0.62–0.69 between the CHU9D and HUI2 and HUI3, with slightly higher correlations for the youth compared to the adult tariff set [24]. The agreement between CHU9D and HUI2 or HUI3 was greater at higher utilities [24]. That research indicated that the CHU9D Sleep domain, a domain not present in the HUI2 or HUI3, had the lowest domain score when youth tariffs were used but Pain was scored lowest with the adult tariff set. The Pain domain score also ranked lowest for the HUI2 and HUI3. The difference between CHU9D youth and adult tariffs with regard to which domain ranked lowest suggests that youth and adults may place more weight on different attributes [38, 53]. Not surprisingly, the highest correlations between the CHU9D and HUI2 or HUI3 were observed for Pain [24]. Pediatric IBD patients can experience pain and this common domain may have contributed to the ability of CHU9D as well HUI2 and HUI3 to distinguish between levels of disease activity in the present study. This may not be the case for other pediatric conditions. The present study demonstrated that compared to the HUI2, the HUI3 generally demonstrated lower and a wider range of mean and median utilities across activity levels, possibly due to different domains, which may account for its superior ability to distinguish between quiescent and mild disease activity levels. Although differences in utilities between instruments were mostly small, they could impact QALY calculations. A probabilistic analysis that integrates ranges of utilities for a given heath states corresponding to the values observed in this study is recommended.

Although the CHU9D was initially developed with utility weights obtained from adults, an adolescent tariff set was created from a sample of Australian adolescents using a best–worst scaling method [23]. Ratcliffe et al. found that adults placed less weight on mental health impairments (worried, sad, annoyed) and more weight on moderate to severe levels of pain compared to adolescents. Whereas youth tariffs may be more reflective of the adolescent experience and childhood in general compared to adult tariffs, IBD and other chronic pediatric conditions have ages of onset less than ten years of age. Choosing a valuation method that can be used in children to generate underlying weights remains a challenge [14]. At present, guidelines for economic evaluation prefer utilities elicited using a SG or TTO approach [54, 55]. As pressure increases to conduct cost-utility analysis to inform budget allocation for drugs and technologies for children, guidelines will need to reflect newer methods and approaches for eliciting and deriving utilities in children.

The present results can be used to populate health state transition models for cost-utility analysis comparing pediatric IBD treatments, including biologics and less costly biosimilars. Health state transition (Markov) models incorporate the probability of transitioning between health states characterized by different levels of disease activity or severity [56, 57]. The effectiveness of different treatments can be compared by capturing how much time is spent in a given health state (e.g., inactive versus active) over the designated study time horizon. For example, from the present analysis, a CHU9D youth utility of 0.81 (SD 0.17) can be assigned to a disease remission (inactive) health state and 0.69 (SD 0.21) would be assigned to a relapse (active) state in CD [58]. More effective treatments will result in children spending more time in the higher utility inactive state. The better that active disease states can be distinguished from inactive states in terms of utility, the more sensitive an analysis will be to true differences in treatment effectiveness. Thus preference-based HRQOL measures that are better at discriminating between active and inactive disease will be stronger options for conducting cost-effectiveness analysis compared to instruments that are less discriminant.

Including multiple clinical disease activity scales and categorizations, preference-based instruments, and tariff sets in the present study provide researchers with the opportunity to choose utilities that align best with their target study population, preferred health economic protocols, and clinical practice. Further, the present study indicates that in the absence of detailed objective clinical data for the complete scoring of the wPCDAI or PUCAI, a PGA may provide reliable utility estimates across disease activity levels. The similarities in utilities within HRQOL instruments when disease activity is defined by the PGA or wPCDAI in CD and by the PGA or PUCAI in UC suggest that QALY calculations will not be affected and economic evaluations conducted using utilities associated with disease activity described by any of these clinical scales would be comparable. Despite uncertainty arising from variation across instruments and tariff sets, establishing genuine pediatric health utilities in CD and UC addresses an important gap.

While a strength was comparing different pediatric utility instruments within the same groups of patients, the study did not include alternative approaches such as TTO, visual analog scales, or discrete choice options to generate utilities. These approaches can be challenging to administer in children. Another strength was the comparison of utilities between CD and UC populations at the same clinical sites, thereby controlling for variations in practice patterns or standards of care. However, enrollment was limited to three Canadian centres which may limit generalizability. The need to examine sub-groups according to ethnic diversity and gender is growing in importance. The present analysis found that utilities were significantly lower in females with UC as measured by the CHU9D. Such a finding has not been reported previously in IBD except in adult CD patients with utility assessed using the SF-6D [59]. An important limitation was the small samples in some of the disease activity levels. A large proportion of participants in this observational cohort were well managed and in a quiescent state, and few had severe disease activity. This hampered the comparison of utilities between quiescent, mild, moderate and severe activity states. However, when dichotomized into active and inactive disease, there was sufficient statistical power to detect a significant difference in utilities in CD and in UC with all instruments. Another limitation was the skewness of the data. It’s important to consider median utilities and IQRs alongside means and standard deviations to interpret the findings.

In conclusion, the CHU9D calculated with adult and youth tariffs, the HUI2 and the HUI3 discriminated between levels of disease activity experienced by children with CD and UC from quiescent to severe disease activity. These utilities may be valuable for health state transition models assessing the cost-effectiveness of emerging treatments in pediatric IBD.

## Supplementary Information

Below is the link to the electronic supplementary material.

Supplementary file1 (PDF 131 KB)

## Data Availability

The raw datasets generated during and/or analysed during the current study are not publicly available and are proprietary to The Hospital for Sick Children. Data summaries are available at https://cidscann.ca.
