# Supplementary material for: A comparison of the Child Health Utility 9D and the Health Utilities Index for estimating health utilities in pediatric inflammatory bowel disease
Source: Qual Life Res. 2023 Apr 1;32(9):2527–39. doi: 10.1007/s11136-023-03409-x (PMC10393835; doi:10.1007/s11136-023-03409-x)
Supplement: Supplementary file 1 — Supplementary file1 (PDF 131 KB) [file 11136_2023_3409_MOESM1_ESM.pdf]

Online Resource 1: Description of Data Collection Instruments

A Comparison of the Child Health Utility 9D and the Health Utilities Index for Estimating Health Utilities in Pediatric Inflammatory Bowel Disease

Running title: Health state utilities in pediatric IBD

Naazish S. Bashir PhD[1], Thomas D. Walters MBBS, MSc, FRACP[2], Anne M. Griffiths MD, FRCP(C)[2], Anthony Otley, MD, MSc, FRCPC[3], Jeff Critch MD, FRCPC[4], Wendy J. Ungar MSc, PhD[1,5]

Author affiliations:

1. Program of Child Health Evaluative Sciences, The Hospital for Sick Children Research Institute, Toronto, Ontario, Canada
2. Division of Gastroenterology, Hepatology and Nutrition, The Hospital for Sick Children, Toronto, Ontario, Canada, and Department of Paediatrics, University of Toronto, Toronto, Ontario, Canada
3. Departments of Paediatrics and Medicine, Dalhousie University, Halifax, Nova Scotia, Canada and Division of Gastroenterology & Nutrition, IWK Health Centre, Halifax, Nova Scotia, Canada
4. Department of Pediatrics, Janeway Children's Health and Rehabilitation Centre, and Faculty of Medicine, Memorial University, St. John's, Newfoundland, Canada
5. Institute of Health Policy, Management and Evaluation, University of Toronto, Toronto, Ontario, Canada.

Correspondence: Dr. Wendy J. Ungar, The Hospital for Sick Children, Peter Gilgan Centre for Research and Learning, 686 Bay Street, 11th Floor, Toronto, ON M5G 0A4. E-mail: wendy.ungar@sickkids.ca Tel: (416) 813-8519; Fax: (416) 813-5979;
ORCID: 0000-0002-0762-0101

Description of Data Collection Instruments

This research used two generic preference-based HRQOL questionnaires to elicit health utilities in children with UC and CD: the Child Health Utility 9D (CHU9D) and Health Utilities Index (HUI). The CHU9D was developed in 2009 specifically for children and with children [1; 2]. It features a descriptive classification system of health states relevant to child health with nine dimensions: Worried, Sad, Pain, Tired, Annoyed, Schoolwork, Sleep, Daily routine, and Activities [3; 4]. Each dimension can be described by five levels. Utility weights, or tariffs, for health states described by the system were obtained from samples of Australian adults or from Australian adolescents using a best-worst scaling method [5; 6]. As part of the present research program, the validity of the CHU9D in children with IBD was previously evaluated [7].

The HUI generates health utilities using the HUI Mark 2 (HUI2) or the HUI Mark 3 (HUI3) which are multi-attribute health classification systems [8]. The HUI has been validated for use in children as young as 8 years and with parent proxies for children over 5 years. The HUI2 has 7 dimensions: Sensation, Mobility, Emotion, Cognition, Self-Care, Pain and Fertility, and the HUI3 has 8 dimensions: Vision, Hearing, Speech, Ambulation, Dexterity, Emotion, Cognition and Pain [8; 9]. The HUI2 has 3 to 5 levels of ability/disability and the HUI3 has 5 or 6 levels of ability/disability [9]. With different valuation algorithms, single and multi-attribute utilities can be calculated for the HUI2 and HUI3 using the same HUI questionnaire in children [9]. The HUI has been used in numerous patient populations and across several age groups [9], but had not been used in pediatric IBD [10].

In children with CD, the Pediatric Crohn's Disease Activity Index (PCDAI) is a standardized method of assessing disease activity [11]. The weighted Pediatric Crohn's Disease Activity Index (wPCDAI), a shorter form, has become widely accepted to assess disease activity in children with CD [11; 12]. Numerical, non-continuous scores correspond to health categories of remission (quiescent), mild, moderate and severe disease activity [11]. Similarly, the Pediatric Ulcerative Colitis Activity Index (PUCAI) is an accepted tool to assess disease activity in children with UC and categorizes UC as remission (quiescent), mild, moderate, and severe [13-15]. Both the wPCDAI and PUCAI have been well correlated with the Physician Global Assessment (PGA) [11; 15; 16]. The PGA rating is based on the physician's determination of a patient's health and unlike the PCDAI and the PUCAI, may not take into account lab values or other test results, but may be more readily attainable [11; 15]. The PGA describes disease activity as quiescent or none, mild, moderate, severe, or fulminant.

References

1. Stevens, K. (2009). Developing a descriptive system for a new preference-based measure of health-related quality of life for children. *Quality of Life Research, 18*(8), 1105-1113. doi:http://dx.doi.org/10.1007/s11136-009-9524-9

2. Stevens, K. (2011). Assessing the performance of a new generic measure of health-related quality of life for children and refining it for use in health state valuation. *Applied Health Economics and Health Policy, 9*(3), 1-13.

3. Stevens, K., & Ratcliffe, J. (2012). Measuring and valuing health benefits for economic evaluation in adolescence: an assessment of the practicality and validity of the Child Health Utility 9D in the Australian adolescent population. *Value in Health, 15*(8), 1092-1099. doi:https://doi.org/10.1016/j.jval.2012.07.011

4. Stevens, K. J. (2010). The Child Health Utility 9D (CHU9D). A new, paediatric, preference-based measure of health related quality of life. *PRO Newsletter, 43*, 11-12.

5. Ratcliffe, J., Huynh, E., Stevens, K., Brazier, J., Sawyer, M., & Flynn, T. (2016). Nothing About Us Without Us? A Comparison of Adolescent and Adult Health-State Values for the Child Health Utility-9D Using Profile Case Best-Worst Scaling. *Health Economics, 25*(4), 486-496. doi:10.1002/hec.3165

6. Ratcliffe, J., Huynh, E., Chen, G., Stevens, K., Swait, J., Brazier, J., Sawyer, M., Roberts, R., & Flynn, T. (2016). Valuing the Child Health Utility 9D: Using profile case best worst scaling methods to develop a new adolescent specific scoring algorithm. *Social Science & Medicine, 157*, 48-59. doi:10.1016/j.socscimed.2016.03.042

7. Bashir, N. S., Walters, T. D., Griffiths, A. M., & Ungar, W. J. (2021). An assessment of the validity and reliability of the pediatric Child Health Utility 9D in children with Inflammatory Bowel Disease. *Children, 8*(5), 343.

8. Horsmann, J., Furlong, W., Feeny, D., & Torrance, G. (2003). The Health Utilities Index (HUI): concepts, measurement properties and applications. *Health and Quality of Life Outcomes, 1:54*. doi:doi:10.1186/1477-7525-1-54

9. Horsmann, J., & Gauld, M. (2018). Health Utilities Inc. Retrieved from http://healthutilities.com/

10. Knowles, S. R., Graff, L. A., Wilding, H., Hewitt, C., Keefer, L., & Mikocka-Walus, A. (2018). Quality of life in Inflammatory Bowel Disease: A systematic review and meta-analyses—Part I. *Inflammatory Bowel Diseases, 24*(4), 742-751. doi:10.1093/ibd/izx100

11. Turner, D., Griffiths, A. M., Walters, T. D., Seah, T., Markowitz, J., Pfefferkorn, M., Keljo, D., Waxman, J., Otley, A., & LeLeiko, N. S. (2012). Mathematical weighting of the pediatric Crohn's disease activity index (PCDAI) and comparison with its other short versions. *Inflammatory Bowel Diseases, 18*(1), 55-62. doi:https://doi.org/10.1002/ibd.21649

12. Mack, D. R., Benchimol, E. I., Critch, J., deBruyn, J., Tse, F., Moayyedi, P., Church, P., Deslandres, C., El-Matary, W., Huynh, H., Jantchou, P., Lawrence, S., Otley, A., Sherlock, M., Walters, T., Kappelman, M. D., Sadowski, D., Marshall, J. K., & Griffiths, A. (2019). Canadian Association of Gastroenterology clinical practice guideline for the medical management of pediatric luminal Crohn’s Disease. *Journal of the Canadian Association of Gastroenterology, 2*(3), e35-e63. doi:10.1093/jcag/gwz018

13. Turner, D., Levine, A., Escher, J. C., Griffiths, A. M., Russell, R. K., Dignass, A., Dias, J. A., Bronsky, J., Braegger, C. P., & Cucchiara, S. (2012). Management of pediatric ulcerative colitis: joint ECCO and ESPGHAN evidence-based consensus guidelines. *Journal of Pediatric Gastroenterology and Nutrition, 55*(3), 340-361.

14. Turner, D., Otley, A. R., Mack, D., Hyams, J., De Bruijne, J., Uusoue, K., Walters, T. D., Zachos, M., Mamula, P., Beaton, D. E., Steinhart, A. H., & Griffiths, A. M. (2007). Development, validation, and evaluation of a pediatric Ulcerative Colitis Activity Index: A prospective multicenter study. *Gastroenterology, 133*(2), 423-432. doi:10.1053/j.gastro.2007.05.029

15. Turner, D., Hyams, J., Markowitz, J., Lerer, T., Mack, D. R., Evans, J., Pfefferkorn, M., Rosh, J., Kay, M., Crandall, W., Keljo, D., Otley, A. R., Kugathasan, S., Carvalho, R., Oliva-Hemker, M., Langton, C., Mamula, P., Bousvaros, A., Leleiko, N., et al. (2009). Appraisal of the pediatric ulcerative colitis activity index (PUCAI). *Inflammatory Bowel Diseases, 15*(8), 1218-1223. doi:10.1002/ibd.20867

16. Turner, D., Griffiths, A. M., Mack, D., Otley, A. R., Seow, C. H., Steinhart, A. H., Silverberg, M. S., Hyams, J., & Guyatt, G. H. (2010). Assessing disease activity in ulcerative colitis: patients or their physicians? *Inflammatory Bowel Diseases, 16*(4), 651-656. doi:10.1002/ibd.21088
